# *Listeria Monocytogenes* Biofilm Removal Using Different Commercial Cleaning Agents

**DOI:** 10.3390/molecules25040792

**Published:** 2020-02-12

**Authors:** Annette Fagerlund, Even Heir, Trond Møretrø, Solveig Langsrud

**Affiliations:** Nofima, Norwegian Institute of Food, Fisheries and Aquaculture Research, 1433 Ås, Norway; annette.fagerlund@nofima.no (A.F.); even.heir@nofima.no (E.H.); trond.moretro@nofima.no (T.M.)

**Keywords:** cleaning, disinfection, biofilm, *Listeria monocytogenes*, food safety, enzymatic cleaning

## Abstract

Effective cleaning and disinfection (C&D) is pivotal for the control of *Listeria monocytogenes* in food processing environments. Bacteria in biofilms are protected from biocidal action, and effective strategies for the prevention and removal of biofilms are needed. In this study, different C&D biofilm control strategies on pre-formed *L. monocytogenes* biofilms on a conveyor belt material were evaluated and compared to the effect of a conventional chlorinated, alkaline cleaner (agent A). Bacterial reductions up to 1.8 log were obtained in biofilms exposed to daily C&D cycles with normal user concentrations of alkaline, acidic, or enzymatic cleaning agents, followed by disinfection using peracetic acid. No significant differences in bactericidal effects between the treatments were observed. Seven-day-old biofilms were more tolerant to C&D than four-day-old biofilms. Attempts to optimize biofilm eradication protocols for four alkaline, two acidic, and one enzymatic cleaning agent, in accordance with the manufacturers’ recommendations, were evaluated. Increased concentrations, the number of subsequent treatments, the exposure times, and the temperatures of the C&D agents provided between 4.0 and >5.5 log reductions in colony forming units (CFU) for seven-day-old *L. monocytogenes* biofilms. Enhanced protocols of conventional and enzymatic C&D protocols have the potential for improved biofilm control, although further optimizations and evaluations are needed.

## 1. Introduction

Cleaning and disinfection (C&D) in food industry premises are important to ensure microbial food quality and safety. The cleaning process removes food residues, soils, and organic matter that accumulate on surfaces during the production. In some cases, a bactericidal effect is also achieved [1]. Most commonly, alkaline foam or gel cleaning agents are used for the open cleaning of processing equipment, walls, and floors. The caustics act through the solubilization, swelling, and degradation of food soils, which aids the removal of soils from the surfaces. The cleaning agents may also contain additives such as surfactants, sequestrants, and hypochlorite, which improve wetting, degradation, and the removal of soils [2,3,4]. Acidic foam cleaners are used occasionally by some companies to remove mineral deposits. Fluid-processing equipment (e.g., pasteurizers) is cleaned with non-foaming alkaline and/or acidic agents, which are often used at higher concentrations and temperatures than employed during open cleaning. After cleaning, food contact surfaces (and often also floors and walls) are usually disinfected to further reduce the number of microorganisms to obtain a level that ensures the production of safe food that meets the expected shelf life [5].

The pathogen *Listeria monocytogenes* is frequently found to persist in food processing plants despite thorough and regular C&D [6,7,8]. It has been shown to persist in specific niches, and drains, slicers, and conveyors are common harborage sites [9,10]. The exact reason for this persistence is not clear, and it could be linked to both properties of the bacterium itself (e.g., growth at low temperatures, specific disinfectant resistance mechanisms, and biofilm production) and to the niches where it is most commonly found (difficult to reach by C&D, humid, collecting nutrients) [6,8]. Nevertheless, sanitation is used both as a preventive and corrective action [11,12,13] in *Listeria* control programs. It is recommended to apply the recommended user concentrations, temperatures, and times set by manufacturers of C&D agents, but this approach may not be sufficient to eradicate *L. monocytogenes*. Fagerlund et al. [14] showed limited effects of cleaning with a regular alkaline cleaning agent followed by disinfection (several common disinfectants were applied) on *L. monocytogenes* established in mono- and multispecies biofilms on conveyor belts. The belt material was designed with a relatively smooth polyvinyl chloride (PVC) top layer, while the underside was an impregnated woven polyester fabric, and *L. monocytogenes* seemed to be trapped and protected in biofilms within the fibers on the underside of the belt. Similarly, Chaturongkasumrit et al. [15] showed that it was difficult to eliminate *L. monocytogenes* biofilms growing on a polyurethane conveyor belt material by cleaning with an alkaline foam followed by disinfection with Tego-51 (an amphoteric disinfectant). The efficacy was poorer on worn belt materials, which had rougher surfaces. They showed that a slightly better reduction could be obtained by doubling the concentrations of chemicals, but a total eradication of *L. monocytogenes* was not achievable through a regular C&D regime. Indeed, most studies have found that regular C&D agents are not able to remove neither laboratory nor industrial biofilms without applying shear stress, elevated temperatures, and/or an increased holding time (see e.g., [1,16,17,18,19,20]). However, there are some exceptions. For example, Reynisson et al. [21] studied laboratory biofilms produced by a fish bacterial flora and concluded that the biofilms were eliminated under regular C&D concentrations and temperatures, and they suggested that the industry could reduce concentrations.

In a «seek & destroy» strategy to combat *L. monocytogenes* in the food production environment, it has been suggested to use heat treatment of whole equipment or parts of it to eliminate *L. monocytogenes*, when regular C&D after dismantling is not sufficient [12,13]. Other alternatives were to sanitize with 70% alcohol or high concentrations of quaternary ammonium compounds (QAC) without subsequent rinsing. There are several reasons for the industry not implementing these strategies: Heat treatment is often not possible for practical reasons, ethanol cannot be used extensively for health and safety reasons, and concerns about resistance buildup or QAC transfer to food products are expressed among food producers. Another drawback of these approaches is that the soils and nutrients are not removed, and niches where *L. monocytogenes* can establish are therefore maintained. Enzymatic cleaning agents are available on the market and could be an option to regular alkaline foams to remove residual soil, but they are still not commonly used [22,23]. Most enzymes suggested or used as antibiofilm agents target the polymeric substances of the extracellular matrix of bacteria (see e.g., [24,25,26,27,28,29,30]). Therefore, the detergents and enzymes in these cleaning agents are likely to create a disruption and dispersal of biofilms that may cause more effective access for disinfectants and provide potent biofilm eradication and microbial control.

Compared with disinfectants, comparative studies on cleaning agents for removing *L. monocytogenes* biofilms are few, and as far as we know, the effect of commercial cleaning agents has not been investigated. Furthermore, manufacturers of C&D agents recommend approaches to remove biofilms that have not been documented in systematic studies. The aim of this work was to compare the efficacy of different recommended cleaning approaches to remove established *L. monocytogenes* biofilms. To simulate a worst-case scenario, we employed the same conveyor belt *L. monocytogenes* biofilm model system as described in Fagerlund et al. [14], where a common niche for *L. monocytogenes* is combined with *L. monocytogenes* strains that have shown persistence in the food industry, and *L. monocytogenes* were allowed to form biofilms for four days prior to C&D. A typical «standard» cleaning process was compared with cleaning processes recommended for biofilm removal and curative treatment. Protocols and in-use concentrations of the cleaning agents were in accordance with the manufacturer’s suggestions. A disinfection step with peracetic acid was included to simulate a complete C&D process.

## 2. Materials and Methods

### 2.1. Bacterial Strains and Growth Conditions

A mixture of seven *L. monocytogenes* strains, belonging to different phylogenetic clusters, were used as inoculum in the current study: *L. monocytogenes* strains (MLST sequence types in parenthesis): MF4536 (ST9), MF5376 (ST7), MF5634 (ST121), MF5377 (ST8), MF4565 (ST18), MF5630 (ST19), and MF5378 (ST394). The selected strains were the same strains as those previously used [14], which were originally isolated by Møretrø et al. [31].

Bacteria were grown in brain heart infusion (BHI) broth (Oxoid, Basingstoke, UK). Overnight cultures were grown in 5 mL volumes in culture tubes at 30 °C with shaking. All biofilm and growth experiments were carried out at 12 °C. For plating, RAPID’*L. mono* (RLM) agar plates (Bio-Rad, Oslo, Norway) and BHI agar (Oxoid, Basingstoke, UK) plates were used.

### 2.2. Cleaning and Disinfection (C&D) Agents

The selected chemical cleaning agents were purchased from manufacturers in Norway with high market share and are listed in Table 1. The enzymatic cleaning agent was from another European company. The companies provided information about the following: (i) Selection of cleaning agent for regular use (cleaning agent A); (ii) Selection of cleaning agents for removal of *L. monocytogenes* biofilms (B1, B2, C2, Enzymatic agent); and (iii) Selection of cleaning agents and time/concentration combinations for curative cleaning (B1, B2, C1, C2, C3, Enzymatic agent). The agents were used at the minimum recommended user concentrations, unless otherwise specified. All manufacturers recommended the dismantling of equipment and thorough mechanical scrubbing, as well as relatively high temperatures (45 °C or higher) for curative cleaning. All cleaning agents were freshly prepared before use. The ethanol solution was not recommended as a cleaning agent but was included, because some food processing factories use it to clean/disinfect conveyor belts before or after breaks.

The industrial disinfectant agent used was based on peracetic acid and is referred to as «PAA». When examining normal user concentrations of sanitation agents, PAA was used at the indicated minimum user concentration, 1.5%, at which the solution contains a minimum of 0.02% peracetic acid, 0.05% acetic acid, and 0.15% hydrogen peroxide. When testing potential curative treatments (see Section 2.5.3), PAA was used at 3% concentration as the elevated disinfectant concentration, as recommended by the manufacturers.

### 2.3. Bactericidal Suspension Tests

Overnight cultures of the seven tested *L. monocytogenes* strains were mixed in equal numbers, and the combined suspension was diluted to approximately 10^8^ CFU ml^−1^ in peptone water. One ml of the diluted culture was added directly to 9 mL of deionized H_2_O (control) or user concentrations of cleaning agents (Table 1), resulting in a final cell concentration of approximately 10^7^ CFU ml^−1^. After 5 min, 0.5 mL of the suspension was transferred to 4.5 mL Dey–Engley (D/E) neutralizing broth (Difco, New Jersey, USA), and dilutions were plated on BHI agar plates. The tests were performed with all solutions at 12 °C. The experiment was performed twice.

### 2.4. Conveyor Belt Biofilm Assay

Biofilms of *L. monocytogenes* were produced and harvested as described in Fagerlund et al. [14]. In brief: Overnight cultures of the seven *L. monocytogenes* strains were mixed in equal volumes and diluted to a final concentration of ~10^6^ CFU ml^−1^. Inoculums of 1 mL were used to inoculate each coupon (1.0 × 1.5 cm) of food-grade PVC conveyor belt material (E8/2 U0/V5 MT white FDA, Forbo-Siegling Transilon, Baar, Switzerland) placed vertically in 24-well plates, so that the air/liquid interface crossed the length of the coupon. Biofilms were grown at 12 °C for 4 days before C&D experiments were commenced. Then, coupons were treated with C&D on days 4, 5, 6, and 7, as detailed in Section 2.5, and they were incubated in fresh BHI at 12 °C between treatments. The harvest of coupons was performed before and after coupons were subjected to C&D on the first and/or last days of C&D treatment (days 4 and 7), as described [14]: After rinsing three times in H_2_O to remove nonadherent bacteria, coupons were vortexed with glass beads and sonicated (40kHz, 10 min, Branson 3510, Bransonic Ultrasonic Corporation, Soest, The Netherlands). Then, dilutions of suspended bacteria were plated on BHI or RLM agar plates and incubated for 2 to 3 days at 30 °C and 37 °C, respectively.

### 2.5. C&D Treatment of Biofilms

**Figure 1:**
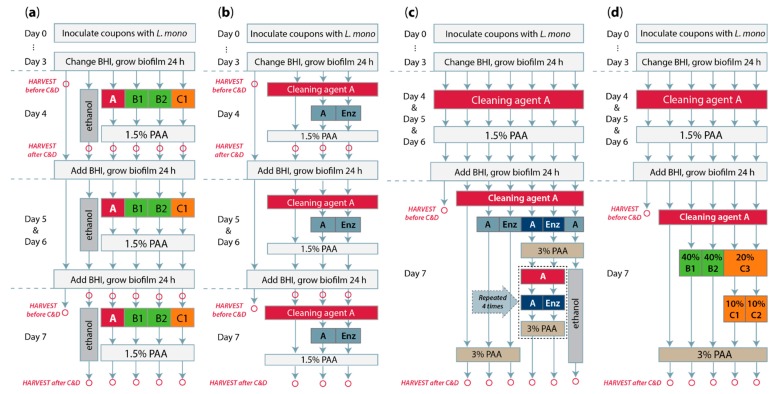
Diagrams illustrating the applied cleaning and disinfection (C&D) treatments. Each arrow represents the step in the protocol where coupons were rinsed three times in H_2_O. Red circles indicate the steps where coupons were harvested for the determination of colony forming units (CFU) coupon^−1^. For steps involving cleaning agent A, red boxes indicate incubation for 10 min at room temperature (RT), while blue boxes indicate incubation for 30 min with cleaning solution pre-heated to 45 °C, and incubation either at (**b**) RT or (**c**) 45 °C. For additional details, see the main text. (**a**) Standard C&D treatment described in Section 2.5.1 and employed in the experiment shown in Figure 2. (**b**) C&D biofilm treatment with the enzymatic cleaning agent described in Section 2.5.2 and Figure 3. (**c**) Reinforced treatment with cleaning agents, see Section 2.5.3 and Figure 4a. The cleaning steps shown in light blue and dark blue boxes («A» and «Enz») represent identical treatments; different coloring (light/dark blue) is employed to coordinate with the colors used in Figure 4a. (**d**) C&D with higher concentrations of chemical cleaners, see Section 2.5.3 and Figure 4b.

The different C&D treatments employed in the current study are illustrated in Figure 1.

#### 2.5.1. Standard C&D Biofilm Treatment

**Figure 2:**
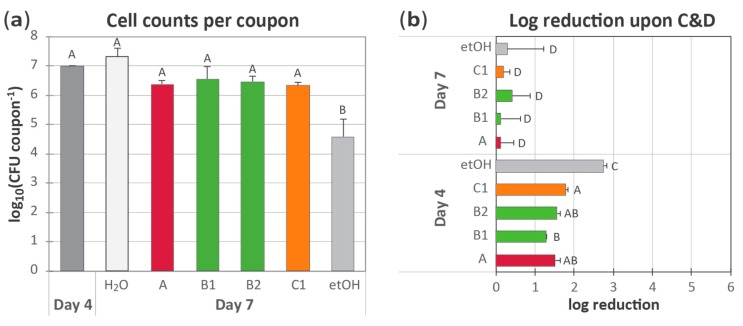
Biofilms of *L. monocytogenes* were allowed to develop undisturbed on conveyor belt coupons until day 4. Then, coupons were treated each day with either (i) rinsing in H_2_O, (ii) cleaning with cleaning agents A (2%), B1 (2%), B2 (3%), or C1 (1%), followed by disinfection with a peracetic acid (PAA)-based disinfection agent (1.5%), or (iii) incubation in 75% ethanol (etOH). (**a**) Total counts of *L. monocytogenes* in conveyor belt biofilms prior to treatments (Figure 1**a**; day 4) and after three consecutive days of treatments followed by 24 h of regrowth in brain heart infusion (BHI) culture medium (Figure 1**a**; day 7). (**b**) Tolerance of biofilms to treatment regimes, shown as the log reductions in bacterial counts upon C&D (or ethanol) treatment on days 4 and 7. For each experiment, the mean values of two replicates with standard errors of the means are shown. Different letters indicate a statistically different effect of treatments (confidence level 95%); in (**b**), data for days 4 and 7 were considered separately in the statistical analysis.

The standard C&D biofilm treatment (Figure 2) was performed as follows: Coupons were rinsed three times in approximately 10 mL of sterile deionized H_2_O (in 15-mL Falcon tubes) to remove nonadherent bacteria and placed vertically in wells of a clean 24-well tray. Then, a chemical cleaning agent (either agent A, B1, B2, or C1) was applied to each well in the form of foam (as intended by the manufacturers; produced in foam pump bottles from Sunvita, Bergen, Norway). The coupons were incubated in cleaning agent for 10 min before coupons were rinsed as before in H_2_O and placed in a second clean 24-well plate. Then, the wells were filled with 1.5% peracetic acid-based disinfection agent (PAA) foam, and the coupons were incubated 5 min and finally rinsed in H_2_O again. Coupons treated in parallel with 75% ethanol were processed as follows: These were subjected to the first and last rinse in H_2_O (as above), but they were incubated in ethanol while the coupons processed in the same experiment were subjected to C&D treatment (10 min in cleaning agent, rinse, 5 min in PAA). Control coupons were rinsed three times with H_2_O every day. The entire experiment was performed at room temperature (RT) (approximately 20 °C).

#### 2.5.2. C&D Biofilm Treatment with Enzymatic Cleaning Agent

**Figure 3:**
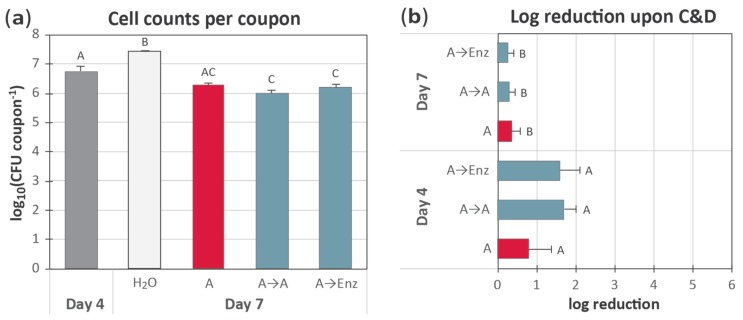
The four-day-old biofilms were subjected to C&D treatments on days 4, 5, 6, and they were allowed 24 h of regrowth before sampling. The C&D treatments were either (i) 2% Agent A for 10 min only (A); (ii) 2% Agent A for 10 min followed by 2% Agent A at 45 °C for 30 min at RT (A→A); or (iii) 2% Agent A for 10 min followed by an Enzymatic Agent pre-heated to 45 °C, for 30 min at RT (A→Enz). After these treatments, all coupons were disinfected with 1.5% PAA. (**a**) Counts of *L. monocytogenes* in conveyor belt biofilms prior to C&D at day 4 and day 7. (**b**) Tolerance of biofilms to treatment regimes. Results are based on three replicates; details are otherwise as described in the legend to Figure 2.

In the experiment where coupons were treated with the enzymatic cleaning agent (Figure 3), referred to as «Enzymatic agent» (Table 1), the protocol was modified as follows: All coupons were treated as described above with cleaning agent A and rinsed in H_2_O. Then, coupons were either left in H_2_O, treated with either 2% agent A or normal user concentrations of Enzymatic agent, both pre-heated to 45 °C and applied as foam. Incubation was performed for 30 min at RT before rinsing and treatment with PAA, as described in Section 2.5.1.

#### 2.5.3. Curative C&D Biofilm Treatment

**Figure 4:**
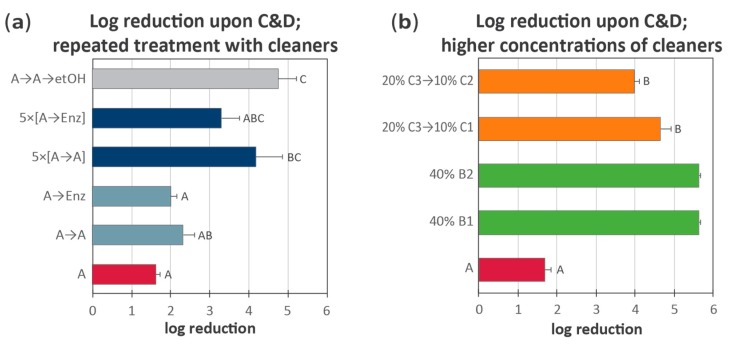
Tolerance of biofilms with reinforced cleaning protocols. Biofilms were allowed to develop for 4 days, and coupons were then cleaned with agent A followed by disinfection with 1.5% PAA for three consecutive days. On day 7, coupons were subjected to different treatment protocols, and the amount of *L. monocytogenes* remaining on coupons after treatment was compared with the number of bacteria present on the day 7 coupons that were subjected only to rinsing in H_2_O. For details on protocols, see the main text and Figure 1c,d. (**a**) Bacterial reductions obtained after biofilm treatment with reinforced cleaning and repeated C&D steps (**b**) Bacterial reductions obtained after biofilm treatment with high dosages of chemical cleaning agents. Experiments in (**a**) and (**b**) were performed at the same time, and the results for the control treatment using standard C&D treatment with cleaning agent A (column A, in red) is shown in both figures. The average CFU per coupon for coupons rinsed in H_2_O only was 1.8 × 10^7^ CFU coupon^−1^, and the detection limit was 5.6 log reductions. Results are based on (**a**) three and (**b**) two biological replicates, each with parallel coupons. Mean values and standard errors of the means are shown, with different letters indicating statistically different effect of treatments (confidence level 95%); except in (**b**), where treatments with agents B1 and B2 reached the detection limit, and therefore, significance was not calculated.

In experiments to test curative treatments, biofilms were allowed to develop for 4 days, as before, and then – on days 4, 5, and 6 – coupons were subjected to treatment with 2% agent A for 10 min followed by 1.5% PAA for 5 min, as described in Section 2.5.1. On day 7, alternative cleaning agent treatments were tested, followed by treatment with PAA at an increased concentration (3%). In the reinforced treatment with the Enzymatic agent (Figure 4a), the same conditions as in the Enzymatic agent assay described above (Section 2.5.2) were used, except that the 30 min incubation step was performed in a closed container at 45 °C, and PAA was used at a concentration of 3%. Both one round and five successive rounds of the protocol were performed, as well as one round followed by treatment with 75% ethanol for the duration of the parallel experiment where coupons were subjected to an additional four rounds of the protocol.

In treatments with higher concentrations of cleaners (Figure 4b), the following cleaning steps were compared: Submersion in 40% agent B1 or B2 for 30 min at RT, submersion in 20% agent C3 for 30 min followed by rinsing in H_2_O as before and incubation either in 10% foam of agent C1 or submerged in 10% agent C2. All coupons were finally rinsed and treated with 3% PAA as before.

### 2.6. Statistical Calculations

Estimates for the mean and variance (standard error of mean of two or three biological experiments) for each treatment plotted in the figures were calculated from the log_10_-transformed values of CFU per coupon (or reduction in CFU per coupon). The total counts used were averages of technical replicates, when included. One-way ANOVA and Tukey’s pairwise comparison were used to test for differences between pairs and groups of treatment means. One-sample t-tests were used to test whether log_10_ reductions were significantly different from 0. Statistical tests were performed in Minitab v18.1 (Minitab Ltd, Coventry, England).

## 3. Results and Discussion

### 3.1. Suspension Tests to Examine Tolerance of Planktonic Cells to the Tested Cleaning Agents

A panel of chemical cleaning agents intended for use in the food industry and recommended by their manufacturers for use against biofilms of *L. monocytogenes* were obtained for testing (Table 1). To examine whether the employed *L. monocytogenes* strains had a specific tolerance toward the employed cleaning agents, bactericidal suspension tests were performed on the *L. monocytogenes* mixture, using the recommended minimum user concentrations for each product (Table 2). We have previously shown that the bacterial reductions were 5 log units after the exposure of these *L. monocytogenes* strains to the employed concentration of PAA disinfectant (1.5%) for 5 min at 12 °C [14].

The bacterial reductions were >5 log units after exposure to the tested concentrations of the chlorinated alkaline cleaners (agents A and B2) for 5 min at 12 °C, but they were only between 1 and 2 log units for the strong alkaline cleaner (B1), while the cleaning-in-place (CIP) alkaline cleaner (C3) gave intermediate results. Since the exact composition of the agents is not known, it is not possible to explain the differences, but chlorine likely contributes to the high bactericidal effect observed, although chlorine has less bactericidal activity at high pH [2].

More than 5 log reduction was also obtained for the strong acidic cleaner (C1), but almost no bactericidal activity was found for the regular acidic cleaner (C2), although the pH was similar for both products. The enzymatic agent also showed listericidal effects, although the pH was relatively neutral, and the enzymes used were not expected to show listeridal activity according to the manufacturer’s information. Again, the exact compositions of the cleaners are not known, so the differences may be due to additives.

### 3.2. Application of Chemical Cleaning Agents to Biofilms at Normal In-Use Concentrations

The efficacy of cleaning agents was tested on biofilms formed from a mixture of seven different strains of *L. monocytogenes* on PVC conveyor belt material coupons, as previously described [14]. The efficacy was compared with the conventional chlorinated alkaline cleaning agent (agent A, Table 1), exposure to a 75% ethanol solution, and with merely rinsing the coupons each day in water. Treatments with cleaning agents, which were employed at the recommended user concentrations, were followed by rinsing and treatment with a foaming peracetic acid-based disinfection agent (PAA). In the initial experiment, the conveyor belt biofilm model was used to compare the use of cleaning agent A with two additional alkaline cleaning agents—a strong alkaline cleaner (agent B1), a chlorinated alkaline cleaner (B2)—as well as a strong acidic cleaner (C1) (Table 1). The results are shown in Figure 2.

After the initial 4 days of biofilm development, the cell densities in the *L. monocytogenes* biofilms reached approximately 1 × 10^7^ CFU per coupon (3 cm^2^ surface area) (Figure 2a, bar: day 4). Next, coupons were subjected to daily cycles of C&D, or daily incubation in 75% ethanol, for three days. Control coupons were rinsed with sterile H_2_O every day. Then, coupons were sampled on day 7 after allowing 24 h of regrowth after the last treatment cycle (with C&D, ethanol, or H_2_O, respectively). Treatment with ethanol resulted in significantly lower cell density on each coupon compared with rinsing in H_2_O or treatment with C&D using one of the four tested cleaning agents (*p* ≤ 0.05 for all relevant two-way comparisons) (Figure 2a). Interestingly, the differences in biocidal activity between cleaning agents as shown in the biocidal suspension tests (Table 2) did not seem to matter, as the effect toward the *L. monocytogenes* biofilms was not significantly lower for agent B1 than for the other tested agents. Probably, the main effect was biofilm removal and not a killing effect in biofilms, as biofilms are generally very resistant to biocidal activity [8]. Overall, no significant differences were detected between any of the other conditions tested. Thus, none of the four C&D regimes tested were able to reduce the total amount of *L. monocytogenes* biofilm on the conveyor belt materials present 24 h after the last C&D treatment.

When the total numbers of CFU per coupon before and after C&D (or ethanol) treatment were compared (Figure 2b), the results showed that overall, the treatments had significantly lower effect on day 7 compared with on day 4 (*p* < 0.001). The day 7 coupons had been subjected to three prior days of treatments, while the day 4 coupons had not. Notably, on day 7, none of the C&D (or ethanol) treatments were able to reduce the amount of *L. monocytogenes* biofilm present on the conveyor belt coupons (one-sample t-tests with H_0_: µ = 0 gave *p* ≥ 0.5 for all treatments). However, when incubation in 75% ethanol was applied on untreated biofilms on day 4, almost a 3 log reduction in cell density on the coupons was obtained. This was a significantly increased reduction compared with that obtained with the four tested C&D treatments, which resulted in between 1.3 and 1.8 log reduction in biofilm on the coupons. Thus, the *L. monocytogenes* biofilms became tolerant toward treatment with all tested C&D regimes and with ethanol treatment within the course of this experiment. We previously observed similar results where the initial tolerance and tolerance development observed were not likely to be a result of specific resistance mechanisms but rather a combination of the attributes of the conveyor belt and broad-spectrum mechanisms linked to the biofilm mode of growth [14]. Others have reported from minor to several log reductions of *L. monocytogenes* in biofilms after C&D in conditions reflecting those found in food processing environments [32,33]. Further studies are needed to rule out whether the various tolerance effects observed are due to the age of the biofilm, adaptive responses of *L. monocytogenes* biofilm cells obtained through exposure to the C&D agents, or a combination of both biofilm age, previous exposure to the C&D agents, or other factors.

### 3.3. Examining the Efficacy of Enzyme-Based Cleaning for Biofilm Removal

According to the user instructions, the enzymatic foaming cleaning agent should be applied for 30 min at 45 °C, between the regular C&D step, to remove biofilms. The efficacy of this treatment in the *L. monocytogenes* conveyor belt biofilm model was tested and compared with two treatment protocols employing C&D treatment using only the conventional cleaning agent A (Table 1): In all three protocols, coupons with biofilm were rinsed and cleaned with room-temperature solution of agent A as before (for 10 min at RT), followed by rinsing three times in H_2_O. Then, one coupon was left standing in the last rinse water, while the other two coupons were subjected to a second step of cleaning (30 min): one coupon was treated with the Enzymatic agent, while the other was treated with agent A; both were pre-heated to 45 °C. After rinsing, all three coupons were subjected to disinfection with PAA as described above. Although the optimum temperature for the Enzymatic agent is 45 °C, in practice, the foam-based cleaning of food industrial surfaces and equipment at 45 °C is likely both difficult (as the materials may keep a temperature of 4–12 °C and cool down the foam) and something one would want to avoid (increasing temperatures may enhance microbial growth). Therefore, while the cleaning agents had a temperature of 45 °C at the time of application, the 30 min exposure time was performed at room temperature (~20 °C). As before, the C&D procedure was performed daily for four days starting with four-day-old biofilms of *L. monocytogenes*, and sampling was performed on the first and last days of C&D treatment (days 4 and 7). The results are shown in Figure 3.

All three C&D treatments resulted in significantly lower cell densities on coupons sampled after allowing 24 h of regrowth after the three consecutive C&D cycles on days 4, 5 and 6, compared with coupons that were only rinsed with sterile deionized H_2_O each day (Figure 3a). However, no statistically significant difference could be detected between the conventional C&D treatment and C&D treatments performed with an extra cleaning step employing either enzymatic or conventional cleaner (Figure 3a).

When examining the log reductions obtained by comparing the total numbers of CFU per coupon before and after C&D treatment, we obtained a similar result as in the experiment shown in Figure 2b: All C&D treatments had a significantly lower effect on day 7 compared to the effect on biofilms treated on day 4 of the experiment (*p* = 0.003) (Figure 3b). In addition, as before, on day 7, none of the C&D treatments were able to significantly reduce the amount of *L. monocytogenes* biofilm present on the conveyor belt coupons (*p* ≥ 0.23).

Furthermore, there was no significant difference between treatments observed in this experiment (*p* = 0.4 for day 4 and *p* = 0.9 for day 7). Thus, under these conditions, the enzymatic cleaner containing enzymes targeting polymeric substances (EPS) of the extracellular matrix, and the conventional chlorinated alkaline cleaning agent, which acts through the unspecific degradation, wetting, and solubilization of organic matters, reduced biofilm by approximately one log_10_. To our knowledge, the effect of commercial enzymatic agents on *L. monocytogenes* biofilms has not been reported before. However, the results were in the same range as in two previous studies on biofilm removal using enzymes, resulting in a modest reduction of 1–2 log_10_ reduction [22,34]. As stated in Section 3.2, the literature shows highly variable results for the effect of commercial alkaline cleaners on *L. monocytogenes* biofilms. In conclusion, disregarding the type of cleaning agent, including one extra cleaning step did not result in enhanced removal of the *L. monocytogenes* biofilm, and it appears not to be a solution for biofilm removal.

### 3.4. Curative C&D Treatments with Extreme Use of Cleaners

The results obtained above (Figure 2; Figure 3) showed that none of the tested C&D regimes were able to significantly reduce the level of bacteria in conveyor belt biofilms subjected to C&D on the three previous days. It is easy to envision similar situations in a food processing facility in which a biofilm has been established and subsequently, perhaps in the face of contamination problems, is subjected to daily C&D without obtaining the desired effect. The manufacturers of the cleaning agents were asked what action they would recommend in such a scenario. Suggestions included the application of repeated cleaning cycles and the use of increased concentrations of the chemical cleaning agents, as well as the use of more than one type of cleaning agent in succession. These suggestions were tested in the current study.

Furthermore, one of the manufacturers stressed that all parts should be allowed to completely dry between each C&D step and that heated water (35–55 °C) should be used for the rinsing steps performed before the application of each cleaning agent. All manufacturers additionally suggested the inclusion of a mechanical brushing step. These approaches were not tested in the current study.

#### 3.4.1. Biofilm Treatment with Reinforced C&D and Repeated C&D Cycles

Although the addition of one extra cleaning step did not seem to significantly increase the removal of *L. monocytogenes* biofilms in our tests (Figure 3), the manufacturer of the enzymatic cleaning agent recommended repeated cleaning cycles for the elimination of *L. monocytogenes* biofilms. Furthermore, they recommended using 45 °C throughout the exposure time for the Enzymatic agent and doubling the concentration of the disinfectant. This approach was tested for one conventional cleaning agent and the Enzymatic cleaning agent, using the following conditions (see Section 2.5.3 and Figure 1c): Biofilm formation was initialized for 4 days as before, followed by treatment of all coupons with the same standard C&D regime on days 4, 5, and 6. This treatment consisted of cleaning with a standard chlorinated alkaline cleaning agent (agent A) followed by PAA disinfectant at 1.5% as previously described. Then, on day 7, coupons were subjected to different C&D protocols, as described in Section 2.5.3 and Figure 1c: (i) A protocol with a standard cleaning step (agent A, 10 min at RT) followed by 3% PAA. (ii) A protocol with the standard cleaning step, followed by reinforced cleaning with either an Enzymatic agent or conventional cleaner (agent A) (30 min with 45 °C during incubation), and finally 3% PAA. (iii) A protocol where the entire C&D cycle [described in ii)] was performed five times in succession. (iv) A protocol where the two-step cleaning cycle with agent A in both steps was followed by the submersion of coupons in 75% ethanol (total time for ethanol exposure approximately 3 hours). The results are shown in Figure 4a.

In this experiment, the highest efficacy was observed for the treatment of coupons that were subjected to two cleaning steps (one for 10 min at RT and one for 30 min at 45 °C) followed by incubation in 75% ethanol (Figure 4a; gray bar). Under these conditions, no bacteria were detected on 50% of the individual tested coupons, and this treatment thus gave about 5 log reduction. In comparison, when a shorter ethanol treatment step was applied without prior cleaning steps and subsequent PAA treatment, 3 log reduction was obtained (Figure 2b; gray bar).

The treatment employing five successive rounds of the reinforced C&D procedure using the standard alkaline cleaning agent A in both cleaning steps gave the second largest reduction. This was the only treatment (beside ethanol) that was significantly different from using regular one-step cleaning with an alkaline foam and gave above 4 log reduction in CFU on coupons (Figure 4a; 5 × [A→A]). In this test, one of six replicates gave a result below the detection limit.

These two treatments were significantly more effective than the control C&D procedure using only one standard cleaning step (Figure 4a; red bar). Five successive rounds of the reinforced C&D procedure using the enzyme-based cleaner in the second cleaning steps (Figure 4a; 5 × [A→Enz]) gave a larger average log reduction than the standard control C&D treatment (Figure 4a; red bar); however, the variation in performance was high, and the difference was not significantly different (*p* = 0.12). Since the cleaning efficacy of alkaline increases with temperature [2], it is tempting to speculate that the increased effect of several repeating exposures to cleaning agent A is merely a result of higher temperature and a longer exposure time to alkaline. For the combination with enzymes, the increase in exposure time and temperature was not enough to result in a significantly increased biofilm removal.

#### 3.4.2. Biofilm Treatment with High Dosages of Chemical Cleaning Agents

The use of highly increased concentrations of chemical cleaning products, along with the soaking of dismantled equipment parts in these solutions for extended periods of time, was suggested by the manufacturers as an approach to eliminate *L. monocytogenes* biofilms. Another advice was to use more than one type of cleaning agent. We selected to test the application of increased concentrations and incubation times of cleaning agents in an attempt to achieve a more efficient removal of biofilm in our model. For an overview of the tested protocols, see Section 2.5.3 and Figure 1d.

For testing the alkaline foaming agents B1 and B2, coupons were submerged for 30 min in 40% solution of each cleaning agent. This concentration is 5–7 times higher than the highest indicated user concentrations when the products are employed in regular cleaning. According to the manufacturer, this strategy had not been tested in situ before, as the cleaners were relatively new. In line with the results from an early study on *L. monocytogenes* biofilm eradication [35], the manufacturer of C1, C2, and C3 had experienced a good effect of increased temperatures, concentrations, and the sequential use of alkali and acid cleaners in problem solving. An initial 30 min soaking step with a 20% solution of an alkaline clean-in-place (CIP) detergent cleaner, agent C3, was used prior to treatment with the acidic cleaner (Table 1). Thus, agent C3 was used at 10 times the normal user concentration for this product. Then, following rinsing, we tested both the cleaning agent C1, applied as foam, and a different non-foaming product intended for CIP cleaning: agent C2 (Table 1). Agents C1 and C2 were used at 10% concentration, which is the highest recommended user concentration, and it was allowed to act for 40 min before rinsing. To be able to compare results across experiments, the conditions were otherwise as described above: Biofilms were developed for four days and then subjected to a standard C&D procedure using agent A and 1.5% PAA on days 4 to 6. After completion of the indicated cleaning steps on day 7, coupons were disinfected with 3% PAA. The results are shown in Figure 4b.

The use of higher cleaning agent concentrations, combinations, and extended exposure times (soaking) of the biofilms to the cleaning agents followed by 3% PAA disinfection showed significant (*p* < 0.003) effects and increased the listericidal effects by between 2 and 4 log reductions compared to the control C&D with 2% agent A and 3% PAA (Figure 4b). The experiment was performed at RT and not at 35–55 °C as the manufacturer recommended, and one would expect an even larger reduction with higher temperature. Arizcun et al. [35] found that the reduction of *L. monocytogenes* biofilm increased by more than 4 logs by increasing the temperature from 20 to 55 °C in a sequential cleaning experiment with alkaline and acetic acid. For the treatment of coupons with 40% solutions of cleaning agents, no bacteria were detected on the tested coupons after C&D treatment, both with the strong alkaline foam gel (B1) and the chlorinated alkaline foam B2 (Figure 4b; green bars) (the detection limit was a log reduction of 5.6).

### 3.5. Overall Comparison of Cleaning Approaches

In the present study, model *L. monocytogenes* biofilms were allowed to develop on a conveyor belt material with a woven underside, which is a well-known niche where *L. monocytogenes* may persist and survive C&D in industrial settings. The obtained results supported the experiences from the industry, as the recommended C&D protocols with conventional and enzymatic cleaning agents showed limited effects against *L. monocytogenes* in biofilms. In addition, repeating the C&D cycle had limited effect, and it can not be regarded as cost effective in a practical situation.

The manufacturers of the C&D agents stressed that mechanical action is needed to remove biofilms, but we chose to not include any significant form of mechanical cleaning and shear forces in the model system. It is likely that higher bacterial reductions would have been observed if scrubbing or rinsing with high-pressure sprays had been applied. However, considering that biofilm formation and bacterial persistence primarily occur on sites and niches in food processing equipment and surfaces where brushing or mechanical forces are not effective due to limited availability or poor hygienic design issues, the model system applied seems relevant. Another factor is the likely higher diversity, complexity, and variations in microbial biofilm structure existing in the food processing environments relative to those reflected in the present study. Here, monospecies biofilms of *L. monocytogenes* formed on a single substrate under defined conditions were used. Biofilms produced by *L. monocytogenes* are complex structures and consist of both polysaccharides, teichoic acid, proteins, and extracellular DNA (eDNA), which is something that may explain why they may be more difficult to eradicate than common food soils or biofilms formed by other bacteria [3,10,14].

High concentrations and extended exposure times of the cleaning and/or disinfection agents were needed to eradicate *L. monocytogenes* biofilms from the conveyor belt in the absence of mechanical action. These conditions were extreme compared to normal in-use conditions, and issues such as an increased use of time, costs for chemicals, health issues, and the sensitivity of equipment and machines to high concentrations of chemicals must be considered before implementing such procedures. An effective alternative to excessive cleaning was using an alcohol disinfectant after a thorough cleaning with a caustic cleaning agent. This approach can be considered as an alternative curative treatment of *L. monocytogenes* house strains.

In addition to the effect on biofilms and removing soil, environmental and health effects also must be taken into consideration when choosing cleaning agents. The exact composition of the commercial agents was not known, and an investigation of the safety of the products is beyond the scope of this paper. However, according to the information provided by the manufacturers in the product safety data sheets, all cleaning agents tested, except for the enzymatic (which were classified as irritating to skin) contained chemicals that cause serious skin and eye damage. The enzymatic agent could cause asthma symptoms. All alkaline cleaning agents were classified as very toxic to aquatic life with long-lasting effects, which was due to their content of alkali, hypochlorite, and/or alkyl amino oxides, while the acidic agents were classified as chemicals with no environmental impact. The enzymatic agent contained an enzyme that was acutely toxic to aquatic organisms. Although the acid and alkaline cleaners in this study showed a similar degree of effect on biofilms, and the latter is more toxic to the environment, one cannot deduce that the food industry should move from alkaline to acidic cleaners in general. For many food processes, the removal of fat or starch is important, and then alkaline cleaners are more effective [3].

## 4. Conclusions

The study supported experiences from the industry showing that *Listeria monocytogenes* biofilms formed on conveyor belt materials are difficult to remove through regular cleaning and disinfection. Both increasing concentrations and combining acidic and alkaline cleaning agents seemed promising for the removal of *L. monocytogenes* biofilms in niches that are difficult to reach for mechanical action. In addition, applying an alcohol disinfectant after thorough cleaning was efficient for eliminating biofilms.

## Figures and Tables

**Table 1:** Cleaning agents used in the current study.

Product	Recommended Use^1^	Intended Use
**Agent A**	2–5%	Chlorinated alkaline foam cleaner for the regular cleaning of open surfaces
**Agent B1**	2–8%	Strong alkaline foam–gel for cleaning heavily soiled surfaces
**Agent B2**	3–6%	Chlorinated alkaline foam for the regular cleaning of open surfaces
**Agent C1**	1–10%	Strong acidic foam cleaner for the removal of protein and mineral salt fouling
**Agent C2**	1–10%	Acidic cleaner for the removal of protein and mineral salt fouling
**Agent C3**	1–2%	Alkaline cleaner for cleaning-in-place (CIP)
**Enzymatic agent**	Two-component (1% + 0.2%), 45 °C	Enzymatic foam cleaner for the prevention and removal of biofilms from open surfaces. Use between regular cleaning and disinfection steps
**75% ethanol**	100%	Ethanol/propanol agent for disinfection of surfaces

^1^According to user information sheets.

**Table 2:** Bactericidal suspension test results (cleaning agents).

Product	Concentration	Bactericidal Suspension Test Results (log reductions)^1^	pH
Agent A	2%	>5.3, >5.4	12.1
Agent B1	2%	1.9, 1.1	12.5
Agent B2	3%	>5.3, >5.4	12.7
Agent C1	1%	>5.3, >5.4	2.0
Agent C2	1%	0.6, 0.5	1.9
Agent C3	1%	3.8, >5.4	12.2
Enzymatic Agent	1% + 0.2%	3.9, 4.0	7.8

^1^ Results from both biological replicates are shown. For the control sample without added cleaning agent, 7.3 and 7.4 log CFU ml^−1^ was obtained.
